# Luminescence lifetime encoding in time-domain flow cytometry

**DOI:** 10.1038/s41598-018-35137-5

**Published:** 2018-11-13

**Authors:** Daniel Kage, Katrin Hoffmann, Marc Wittkamp, Jens Ameskamp, Wolfgang Göhde, Ute Resch-Genger

**Affiliations:** 1 Federal Institute for Materials Research and Testing (BAM), Biophotonics Division 1.2, Richard-Willstätter-Str. 11, D-12489 Berlin, Germany; 2 Quantum Analysis GmbH, Mendelstraße 17, D-48149 Münster, Germany; 3 Department of Physics, Humboldt-Universität zu Berlin, Newtonstr. 15, D-12489 Berlin, Germany

## Abstract

Time-resolved flow cytometry represents an alternative to commonly applied spectral or intensity multiplexing in bioanalytics. At present, the vast majority of the reports on this topic focuses on phase-domain techniques and specific applications. In this report, we present a flow cytometry platform with time-resolved detection based on a compact setup and straightforward time-domain measurements utilizing lifetime-encoded beads with lifetimes in the nanosecond range. We provide general assessment of time-domain flow cytometry and discuss the concept of this platform to address achievable resolution limits, data analysis, and requirements on suitable encoding dyes. Experimental data are complemented by numerical calculations on photon count numbers and impact of noise and measurement time on the obtained lifetime values.

## Introduction

Bioanalytical, diagnostic, and security applications require the fast determination of a steadily increasing number of analytes or events in parallel in a broad variety of detection formats. Among the most popular approaches is the use of multiparametric fluorescence techniques for sample analysis due to their versatility and often straightforward use^1–3^. Many fluorescence techniques rely on a toolbox of luminescent labels for encoding and multiplexing^2–6^. An approved and versatile technique for bioanalytical high-throughput single-particle measurements is flow cytometry (FCM). FCM with optical detection is routinely exploited in the life sciences e.g., for single cell analysis for blood count, in diagnostics as well as for next generation sequencing, here in conjunction with intensity or colour encoded surface-functionalised polymer particles acting as carriers for different DNA sequences. State-of-the-art instruments are capable of reading almost 20 different colour codes^7–9^, but that still does not satisfy the requirements of complex research, e.g. in cell biology and immunology^10^.

Depending on the specific purpose, method development for FCM either faces increasing complexity of analytical problems or the demand for low-cost and robust techniques for routine analyses^11^. Commonly applied spectral multiplexing approaches based on organic fluorophores are limited in both directions: on the one hand, spectral overlap of labels restricts the number of codes^3,12^ and makes elaborate correction schemes necessary. On the other hand, even lower degrees of multiplexing often require sophisticated optical setups with multiple excitation light sources and detection channels which increases instrument complexity and costs. Particularly, II/VI semiconductor quantum dots (QDs) with their characteristic spectral properties allow for the use of a larger number of distinguishable color codes compared to dyes^13,14^ but even their spectral distinction is limited. Furthermore, they are not widely accepted due to possible environmental concerns^15–17^ and their toxicity is still a subject of ongoing discussion^18^.

A promising alternative to spectral multiplexing and intensity encoding is offered by luminescence lifetime as an additional encoding parameter^3,12,18,19^. This provides that luminescence codes could be distinguished based on their characteristic decay kinetics. In combination with spectral encoding, it can increase the number of accessible parameters^20^. Furthermore, due to the availability of fast electronics, miniaturized or portable lifetime measurement setups^21^ can be assembled which allow to complement routine techniques based on spectral encoding by lifetime-encoded ones at reduced cost, especially in countries of the developing world. Lifetime encoding in flow cytometry can find applications ranging from alternative staining strategies for cell analysis to the use of stains or labels whose emission properties are sensitive to their microenvironment or respond to binding events by a change in luminescence decay kinetics. Thereby, it helps to increase the information content from bioassays by efficient detection of multiple analytes or targets in a single measurement.

Lifetime encoding in flow cytometry has been discussed for decades now and increasingly draws interest for high-throughput methods based on time-resolved measurements^4^. However, only very specialized applications^22–25^ have been reported so far and lifetime multiplexing in flow is still not accepted in routine applications due to the limited measurement time per object and the resulting issue of reduced photon count numbers^26,27^. Moreover, reported lifetime detection in flow cytometry mostly relied on phase-domain techniques^24,28–32^, which requires higher signal intensities and might face problems analysing multi-exponential decays. Even though time- and frequency-domain methods are theoretically equivalent in terms of resolution^33^, time-domain methods can be superior for low signal intensities. This can be the case for systems which contain a limited number of emitters like the labelling of biomolecules with low label concentrations or when the excitation intensity has to be limited to avoid photobleaching in multi-step analyses of the same sample. Moreover, time-domain measurements directly visualise the decay kinetics.

In this study, the issues of time-domain cytometry with very short interaction times and limited number of detected photons are addressed. We present a lifetime flow cytometry (LT-FCM) platform based on a compact setup and straightforward time-domain measurements utilizing LT-encoded luminescent beads. These polymer microbeads are loaded with different organic fluorophores exhibiting lifetimes in the nanosecond range or semiconductor quantum dots to extend the accessible lifetime range. The beads serve as a model system in our studies but further applications will not be limited to distinguishing luminescent beads with well-defined properties. We discuss the concept of this platform and address its application potential including achievable resolution limits, data analysis, and requirements on suitable encoding dyes. Experimental data are complemented by numerical calculations on photon count numbers and impact of noise and measurement time on the obtained lifetime values.

## Methods

### Materials

As lifetime encoded systems, we employed commercially available PMMA beads stained with organic dyes from PolyAn GmbH, Germany, and tailor-made melamine beads with a polyelectrolyte-based layer-by-layer (LbL) coating, loaded with CdSe/CdS/ZnS core/shell quantum dots (QDs) and a final poly(sodium 4-styrensulfonate) (PSS) layer provided by the research group of Sukhanova and Nabiev^34,35^. As emitters we chose organic dyes expected to reveal lifetimes in the low nanoseconds range and II/VI semiconductor QDs with lifetimes up to tens of nanoseconds. Table 1 gives a brief overview over the corresponding luminophores of the lifetime-encoded beads as well as the mean bead diameters.

**Table 1:** Lifetime codes and respective luminophores with measurement parameters, mean bead diameter *D* and obtained ensemble (reference) lifetimes calculated as intensity-weighted mean lifetimes from multi-exponential decay fits.

LT code	Luminophore	*D*/μm	*λ*_ex_/nm	Em. range/nm	*λ*_det_/nm	τ/ns
A	Red5 400%	6.5	488	602–800	645 LP	1.72
B	Red/Red5 100:300%	6.3	488	545–800	645 LP	2.71
C	Red 50% (400%*)	9 (6.9*)	488	559–718	590 LP	5.54
D	Pink 400%	6.8	488	528–708	645 LP	7.91
E	CdSe/CdS/ZnS QDs	6.2 ± 0.6	488	621–680	645 LP	22.6

The luminophore designation are trade names of PolyAn GmbH and the percentages denote the loading concentration (*two dye loading concentrations used). Code B is a mixture of codes A and C. Further details on the spectroscopic properties can be found in the Supplementary Information.

### Ensemble fluorescence measurements

Steady state and time-resolved fluorescence measurements have been performed with an FLS920 (Edinburgh Instrument Ltd.) spectrometer using magic angle polarizer settings to prevent artifacts due to rotational motion^36^. Measurements were carried out with standard 1 cm quartz cuvettes (Hellma GmbH & Co. KG) at room temperature (20 °C). The samples are continuously stirred during spectra and decay curve recording to prevent precipitation of beads. For steady state measurements, a xenon lamp has been used as excitation light source. Pulsed excitation for time-resolved measurements was provided by a Fianium Supercontinuum SC400-2-PP (NKT Photonics A/S) laser operated at a repetition rate of 10 MHz (for organic dyes) or 5 MHz (for QD-beads). In time-correlated single photon counting (TCSPC) experiments with an R38090U-50 (Hamamatsu Photonics K.K.) MCP-PMT, a pulse width of the instrument response function (IRF) of about 250 ps at 10 MHz was achieved.

### Flow cytometer design

**Figure 1:**
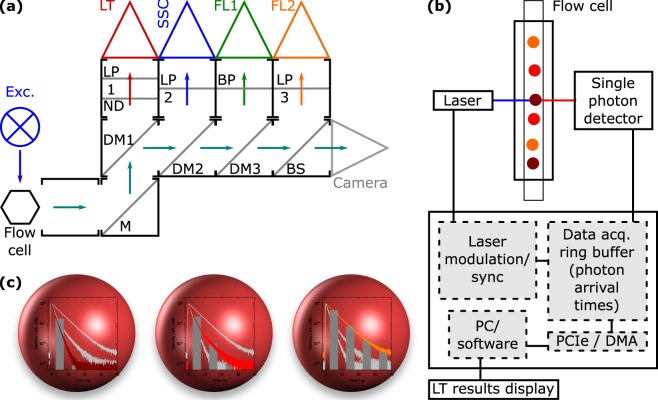
Overview and concept of LT-FCM. (**a**) Schematic drawing of the optical setup of the LT-FCM. Optical components are indicated: (D)M refers to (dichroic) mirrors, BP/LP to band pass/long pass filters, ND to the neutral density filter, and BS to the beam splitter. The detection channels are LT (lifetime), SSC (side scatter), FL1/2 (fluorescence). Details on the optical components can be found in the Supplementary Information, Table S1. (**b**) Block diagram of the novel signal processing unit for single photon lifetime analysis for LT-FCM. (**c**) Visualisation of lifetime encoding with beads: Each code should exhibit similar spectral properties but differ in the decay kinetics from the other codes.

A new lifetime flow cytometer (LT-FCM) including a modulated laser source, novel single photon signal processing lifetime analysis electronics and signal analysis and instrument control software (CyPAD pantau) was designed (Quantum P/pantau, Quantum Analysis GmbH). The new signal processing for FCM is capable of single photon fluorescence lifetime analysis on a time scale of ns and below. This differs significantly from standard FCM, where signals are being integrated within the interaction time of the particle with the laser spot, typically in the μs range. For the LT-FCM, a modulated solid-state laser with associated electronics circuitry based on a 488 nm laser diode (Nichia Corporation) has been designed for excitation (QA MLS Blue pantau). The LT-FCM has been configured with four detection channels, namely ‘LT’: time-resolved single photon fluorescence, ‘SSC’: side scatter, ‘FL1/2’: fluorescence. A schematic drawing and a block diagram of the setup are displayed in Fig. 1(a,b). The laser diode was modulated to yield a 5 MHz square wave output between ‘on’ (100 mW) and ‘off’ (<0.1 mW) with equal periods of time. Average output power was 50 mW. The fall time of the laser power was <800 ps as determined with a photomultiplier tube (PMT) and a 4 GHz/40 GSPS digital oscilloscope (Teledyne LeCroy Waverunner 640Zi). Photon arrival times were detected with a newly designed single photon detector module (QA PM Photodetector Module pantau), based on a H11900 PMT (Hamamatsu Photonics K.K.) and attached to a fast pulse discriminator electronics for single photon pulses <1 ns. The single photon detector was connected to a field-programmable gate array circuitry (FPGA; Altera Cyclone IV) on a newly designed FPGA board with direct connection to a PCI-e slot of a computer board. The FPGA enabled flexible design and (re−)configuration of complex and fast internal digital circuitry (gate delay <200 ps), which significantly helped for optimizations within the development process. The FPGA was programmed with a newly designed digital circuitry to allow photon arrival time measurements, synchronized with the laser modulation, and classification into time bins with a temporal resolution of <2.5 ns per pulse, yielding – at least for the purpose of encoding with selected fluorescent dyes – sufficient sub-ns lifetime analysis resolution with >50–100 single photonic events.

For scattering and fluorescence intensity measurements, conventional PMTs in low-bandwidth (MHz) operating mode were utilised as usual in FCM. Optical components direct spectral fractions of the scattered and emitted light to the respective detectors (see Supplementary Table S1 for details). The sample suspensions have been analysed with flow rates of 1 μl/s.

The interaction time of the encoded beads with the spot of the focused excitation laser was varied by electronically regulating the sheath fluid pressure between 30 and 180 hPa from 4 to 25 μs. The principle of luminescence lifetime encoding with spectrally closely matching luminophores is depicted in Fig. 1(c).

### Lifetime determination

If not stated otherwise, luminescence lifetimes have been calculated from intensity decay curves by means of the well-known Eq. (1)^36^.

1 $${\tau }_{{\rm{mean}}}=\frac{{\int }_{0}^{\theta }\,tI(t){\rm{d}}t}{{\int }_{0}^{\theta }\,I(t){\rm{d}}t}\approx \frac{\sum _{j=1}^{{j}_{{\rm{\max }}}}\,{t}_{j}{I}_{j}}{\sum _{j=1}^{{j}_{{\rm{\max }}}}\,{I}_{j}}$$

Here, *I*(*t*) is the luminescence intensity at time *t*. Strictly, this relation only holds true for *θ* → ∞. Experimental data, however, are clearly limited to finite integration time ranges. Moreover, for discrete data, the integral must be replaced by a sum with finite upper index. The deviation due to missing reconvolution is expected to be negligible since the pulse fall time is much smaller than the bin width (time-resolved flow cytometry). The ensemble lifetimes have been obtained from multi-exponential decay fits involving convolution with the IRF. For clarity, in the following we distinguish between lifetime values based on their origin. Lifetimes obtained from ensemble measurements are referred to as reference lifetimes (since they serve as comparative values in this study), lifetime values used as input for simulations are termed notional lifetimes, lifetimes obtained from our simulations are termed calculated lifetimes and measured lifetimes are those which we obtained with the custom-made LT-FCM.

### Numerical simulation methods

Numerical simulations based on synthetic decay curves were performed. We studied the impact of measurement parameters and conditions such as the integration time range *θ*, the bin width of the decay histograms, the number of acquired photon counts, and the background level. Simulations were carried out with custom-made Octave^37^ scripts. Synthetic decay curves were obtained from random number distributions following exponential decay laws. The generation of these random number distributions was based on computational physics textbook methods^38^. Details are given in the Supplementary Information. Repeated simulations were carried out for each parameter set under study to obtain a reasonable statistical validation of the results. The respective numbers of repetitions are given throughout the discussion and resemble the number of analysed objects in an FCM experiment.

## Results and Discussion

We assessed our newly developed LT-FCM platform described in the Methods Section with a set of microbeads loaded with organic and inorganic luminophores. The overall goal was to evaluate the feasibility and limitations of lifetime encoding in flow with this compact setup. Experimental results were compared with theoretical studies on measurement precision.

### Optical-spectroscopic characterization of lifetime codes

The luminophores used for lifetime encoding had to be excitable at a single common wavelength (here: 488 nm) and detectable within a certain spectral window. Moreover, they should differ in their luminescence decay kinetics for temporal discrimination with poor photon statistics.

**Figure 2:**
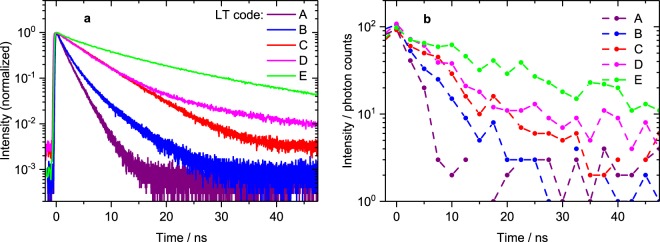
Photoluminescence decay curves of lifetime codes. (**a**) Spectroscopic ensemble measurements: normalised photoluminescence intensity decay curves of lifetime codes for excitation at 488 nm (16 nm band pass; 5 MHz repetition rate for code E, 10 MHz for all others) and detection with a 645 or 590 nm (sample C, for improved signal intensity only) long pass filter. These ensemble measurements demonstrate that the chosen set of encoding beads exhibits significant differences in their decay kinetics and is therefore suitable for the assessment of lifetime encoding in flow cytometry. (**b**) Non-normalised decay curves for single beads of the same samples measured with LT-FCM. The low number of counts per decay curve and the more coarse time resolution underline the challenges compared to ensemble measurements. The dynamic range is much smaller as indicated by the different intensity scales and the obtained lifetime values do not necessarily match those derived from the reference measurements.

Photoluminescence decay curves of bead samples A-E (see Table 1) in ensemble measurements are shown in Fig. 2(a). All luminophores can be excited at 488 nm and the emission (i.e. lifetime code) can be detected with a (broad) band pass or a long pass filter. Fitting of multi-exponential decay functions to the data has been carried out and the intensity-weighted mean lifetimes were calculated as they represent the actual excited state lifetime which is relevant to our studies. The available lifetime codes range from slightly below 2 up to more than 20 ns. Although the decays are multi-exponential due to the inhomogeneous microenvironment of encapsulated luminophores, they are clearly distinguishable. The measurement parameters and mean luminescence lifetimes as well as basic spectroscopic characteristics are summarised in Table 1. Further details on spectroscopic properties are given in the Supplementary Information. Luminescence decay curves of single beads obtained from LT-FCM are displayed in Fig. 2(b). Even though the photon count number and dynamic range are drastically reduced in comparison to the ensemble measurements, the decay kinetics are still distinct.

### Theoretical estimations

Since our measurement technique is based on sparse data and simplified analysis methods, cf. Fig. 2(b) and Eq. (1), we studied the impact of parameters and measurement conditions on the calculated and measured lifetime with simulations and experiments. Even though lifetime encoding approaches do not necessarily require the exact determination of lifetimes but only distinction between different lifetime codes, knowing the limits of the technique is still important. We will discuss the impact of the integration time range used for data analysis, the bin width (channel width) of the luminescence decay histograms, the impact of the number of acquired photon counts and the ratio of signal and background/noise intensity. The latter two are particularly critical in time-resolved flow cytometry due to the short interaction time of each bead or object with the excitation beam.

### Integration time range

**Figure 3:**
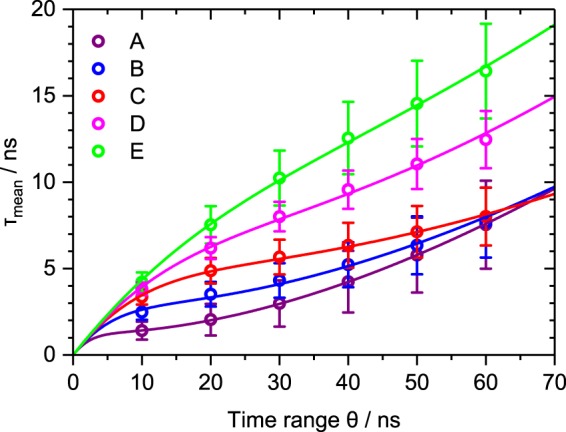
Dependence of the measured lifetime on the integration time range *θ*. Symbols depict averaged results based on LT-FCM data of ≈1000–4000 beads per code. The respective error bars indicate the standard deviation of the resulting lifetime distributions and solid lines are fits using Eq. (5). The choice of *θ* clearly affects the obtained lifetime but it may also be used to improve discrimination.

Starting with the integration time range equivalent to the upper integration limit *θ* in Eq. (1), Fig. 3 highlights the influence of varying *θ* on the measured lifetime. The symbols show measured values derived from data from LT-FCM of different samples. At very short integration time ranges, measured lifetimes are close to zero and not distinguishable. With increasing integration time range, all lifetime values increase. Codes with longer reference lifetimes, however, show a steeper increase of the measured values. This behaviour can be explained by Eq. (2) which results from Eq. (1) assuming a mono-exponential decay with notional lifetime *τ*.

2 $$\begin{array}{rcl}{\tau }_{{\rm{mean}}} & = & \frac{{\int }_{0}^{\theta }\,t{{\rm{e}}}^{-\frac{t}{\tau }}{\rm{d}}t}{{\int }_{0}^{\theta }\,{{\rm{e}}}^{-\frac{t}{\tau }}{\rm{d}}t}\\  & = & \tau -\frac{\theta }{{{\rm{e}}}^{\frac{\theta }{\tau }}-1}\end{array}$$

Considering the derivative with respect to *θ* (not shown), the slope increases with increasing notional lifetime values *τ* as is also seen in the experimental data. Moreover, in the limit of *θ* → 0, we find

3 $$\begin{array}{rcl}\mathop{\mathrm{lim}}\limits_{\theta \to 0}\,{\tau }_{{\rm{mean}}} & = & \mathop{\mathrm{lim}}\limits_{\theta \to 0}(\tau -\frac{\theta }{{{\rm{e}}}^{\frac{\theta }{\tau }}-1})\\  & = & \tau -\mathop{\mathrm{lim}}\limits_{\theta \to 0}\frac{1}{\frac{1}{\tau }{{\rm{e}}}^{\frac{\theta }{\tau }}}=\tau -\tau =0\end{array}$$

which is in accordance with the calculations based on experimental data for values of *θ* close to zero. The opposite limit of *θ* → ∞ leads to

4 $$\mathop{\mathrm{lim}}\limits_{\theta \to \infty }\,{\tau }_{{\rm{mean}}}=\tau $$

suggesting that the measured lifetime approaches the reference lifetime value with increasing *θ*. The convergence of the three shortest lifetime codes for *θ* ≈ 60 ns, however, cannot be described by Eq. (2). Moreover, it gives incorrect off-levelling characteristics for all lifetime codes. Therefore, we added a constant offset *B* to the mono-exponential decay representing background counts and an amplitude *A* to define the ratio of background and noise counts more flexibly (also for multi-exponential decays). The modified version of Eq. (2) then is Eq. (5).

5 $$\begin{array}{rcl}{\tau }_{\mathrm{mean},\mathrm{bg}} & = & \frac{{\int }_{0}^{\theta }\,t(A{{\rm{e}}}^{-\frac{t}{\tau }}+B){\rm{d}}t}{{\int }_{0}^{\theta }\,(A{{\rm{e}}}^{-\frac{t}{\tau }}+B){\rm{d}}t}\\  & = & \frac{A\tau (\tau -[\tau +\theta ]{{\rm{e}}}^{-\frac{\theta }{\tau }})+\frac{B}{2}{\theta }^{2}}{A\tau (1-{{\rm{e}}}^{-\frac{\theta }{\tau }})+B\theta }\end{array}$$

For small *θ*, Eq. (5) can still be approximated by Eq. (2). For $\theta \gg \tau$, a background contribution results in steadily increasing calculated lifetimes for increasing integration time ranges. Figure 3 also displays fits of Eq. (5) to the data (solid lines). These fits follow the course of the data points including the convergence of the three shortest measured lifetime codes despite the simplified assumption of a mono-exponential decay in Eq. (5). The slight indication of saddle points for the three lowest lifetime codes stems from the trade-off between approaching the reference/notional lifetime value for $\theta \gg \tau$ and the steady increase of the measured/calculated lifetime with increasing *θ* due to the background. Thus, lifetime measurements in flow need careful adaption of the integration time range *θ* to ensure good discrimination capabilities and prevent overlap of codes. For multiplexing applications based on lifetime encoding, where the aim is rather distinguishing lifetimes than measuring exact values, the integration time range can be adapted to the specific set of codes focusing only on the code discrimination. The parameters for optimum discrimination can differ from one set of lifetime codes to another one and it also strongly depends on the background count level.

### Bin width

**Figure 4:**
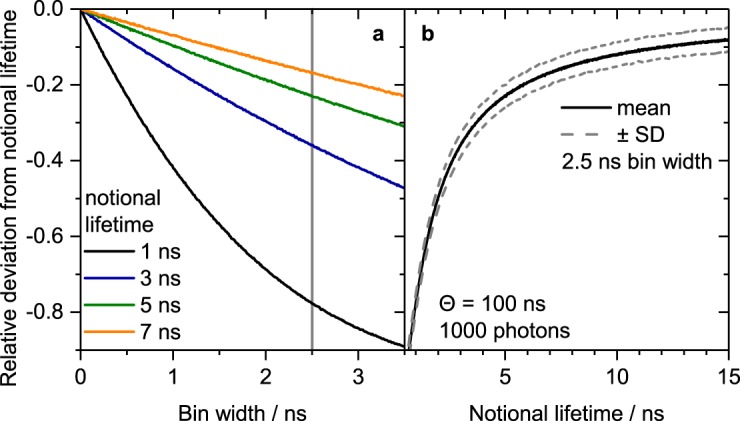
Simulated dependence of the calculated lifetime on (**a**) the bin width and (**b**) the notional lifetime at a fixed bin width. Integration time range was *θ* = 100 ns, background level was zero, 1000 repetitions were done for each parameter set and mean values of the calculated lifetimes are shown. Coarser bin width lead to deviations from the notional lifetimes, especially for short notional lifetimes.

Luminescence decay measurements in the time domain are based on binned data. The simulated dependence of the relative deviation of calculated lifetimes from the notional lifetime on the bin width (channel width) is depicted in Fig. 4(a) for four different assumed notional lifetime values. The calculated lifetime values were obtained from synthetic decay curves generated according to the procedure described in the Methods Section and in the Supplementary Information. The relative deviation shows a drastic decrease of the calculated lifetime values with increasing bin width, i.e. the calculated lifetimes become smaller with increasing bin width. Lifetime codes with shorter lifetimes are more prone to distortion but exhibit smaller standard deviation (*SD*), as is emphasized by Fig. 4(b). For a bin width of 2.5 ns as used in our LT-FCM setup, the deviation for intermediate lifetimes can easily exceed 30% and may approach 100% for short notional lifetimes. The results shown were obtained for using the edges of the bins as time values in Eq. (1). The deviation could be reduced by modified assigned time values (e.g. the middle of each bin) but not completely prevented. This means, lifetime determination with coarsely binned data can distort results such that short reference lifetimes become even shorter whereas longer reference lifetimes are closer to the true values. However, as the intention of lifetime encoding is only the discrimination of lifetime values, this is not a severe drawback. The standard deviation of the calculated lifetime does not change with bin width (cf. Supplementary Fig. S4).

### Photon count number

**Figure 5:**
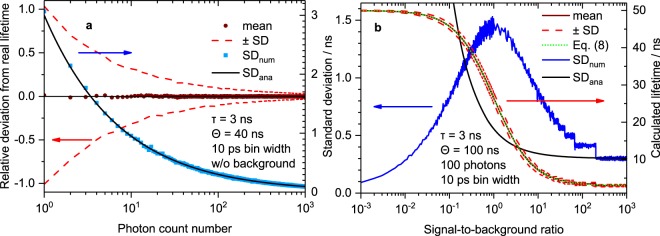
Impact of signal and background intensity. (**a**) Simulated dependence of the lifetime on the number of collected photons. Integration time range *θ* = 40 ns, no background signal, 10 ps bin width. Each data point represents the mean or standard deviation of 1000 repetitions (corresponding to 1000 events in FCM). The mean calculated lifetime turns out to be independent of the photon count number. The standard deviation and therefore the precision benefits from more counts. (**b**) Simulated dependence of the lifetime on the signal-to-background ratio. Integration time range *θ* = 100 ns, 100 photons per event, 1000 repetitions (i.e. events in FCM). The dotted green line represents the analytical expression given by Eq. (8).

Apart from technically determined and directly accessible measurement settings, other key questions in LT-FCM are on the influence of the number of acquired photons per object and the signal-to-noise/background ratio (SNR/SBR) on the measured lifetime values. Thus, we performed simulations with varying number of photons acquired per bead. The dependence of the relative deviation of the calculated lifetime from the notional lifetime on the number of photons without background counts is depicted in Fig. 5(a). The mean calculated luminescence lifetime remains constant and close to the notional value (horizontal black line) over the whole range of photon count numbers studied. However, the numerically determined standard deviation, *SD*_num_, decreases significantly with increasing number of photons, as expected. This means, lifetime measurements could be imprecise at low count numbers, but still accurate. Moreover, there is also no dependence on the photon count number for multi-exponential decays (see Supplementary Fig. S2). Thus, there is no need to restrict luminophores and samples for lifetime encoding to materials with mono-exponential decay. This is an important prerequisite of bead-based lifetime barcodes where heterogeneous microenvironments of fluorophores often lead to multi-exponential decays^39^.

A comparison of the standard deviation, *SD*_num_, numerically determined from our calculations with an analytical expression for mono-exponential decay curve fitting^40,41^, *SD*_ana_, in Fig. 5(a) shows perfect agreement. This emphasizes, that the simple and fast method based on Eq. (1) can compete with more elaborate fitting routines in this case.

### Signal-to-background ratio

In order to get a deeper insight into the impact of background counts on the obtained results, we define the signal-to-background ratio (*SBR*) in Eq. (6).

6 $$SBR=\frac{integrated\,number\,of\,signal\,photons}{integrated\,background\,count\,number}$$

The relationship between the parameters *A* and *B* of the mono-exponential decay with constant offset in Eq. (5) to this *SBR* is given in Eq. (7).

7 $$\frac{A}{B}=\frac{\theta }{\tau (1-{{\rm{e}}}^{-\frac{\theta }{\tau }})}\cdot SBR$$

Combining Eqs (7) and (5) yields Eq. (8) for *B* > 0.

8 $${\tau }_{\mathrm{mean},\mathrm{bg}}=\frac{\frac{SBR}{1-{{\rm{e}}}^{-\frac{\theta }{\tau }}}(\tau -[\tau +\theta ]{{\rm{e}}}^{-\frac{\theta }{\tau }})+\frac{\theta }{2}}{SBR+1}$$

The dependence of the calculated lifetime on the *SBR* is displayed in Fig. 5(b) based on numerical simulations and compared to the analytical expression in Eq. (8). For high *SBR*, the calculated lifetime clearly resembles the notional lifetime. For decreasing *SBR*, the calculated lifetime increases and approaches a constant value. In the limit of *SBR* → 0, Eq. (8) becomes

9 $$\mathop{\mathrm{lim}}\limits_{SBR\to 0}{\tau }_{\mathrm{mean},\mathrm{bg}}=\frac{\theta }{2}$$

which explains the behaviour observed for weak *SBR*. The plot of Eq. (8) given in Fig. 5(b) shows perfect agreement with the numerical simulations. In addition, Fig. 5(b) displays the numerically determined standard deviation, *SD*_num_, of the calculated lifetime values. With decreasing *SBR* the standard deviation increases until it reaches a maximum when photon count number and background count number are equal (*SBR* = 1). With further decreasing *SBR*, the standard deviation decreases again. However, this does not indicate more reliable results, but only a weaker variability of still inaccurate values. Moreover, Fig. 5(b) also shows the theoretical standard deviation for lifetime determination by curve fitting, *SD*_ana_^40^. In contrast to the case without consideration of background shown in Fig. 5(a), the standard deviation from our numerical simulations differs strongly from that expected for curve fitting. This means, the method defined by Eq. (1) is more sensitive to background signal impact and more elaborate methods could improve the analysis.

Based on these results, only the *SBR* is expected to affect the calculated or measured lifetime values once the measurement parameters (integration time range *θ* and bin width) are fixed. The number of acquired photons itself does not influence the obtained lifetime mean values (the standard deviation is, of course, affected).

### Comparison of simulations and experimental results

**Figure 6:**
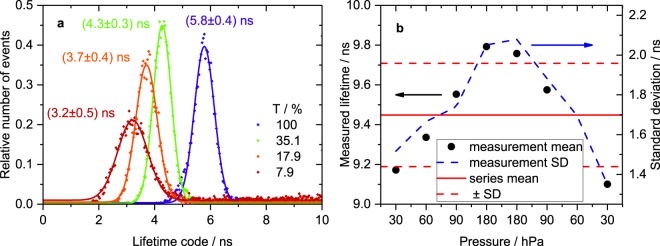
Influence of technical parameters. (**a**) Lifetime distribution of code B obtained from LT-FCM with varying neutral density filter transmittance *T* in the detection channel. Fits of Gaussian distributions have been added for determination of mean values and standard deviations and as guides to the eye. For each transmittance value, ≈8000–12000 beads were measured and the time range was set to *θ* = 20 ns. There is a distinct shift of the distributions towards longer mean lifetimes with increasing filter transmittance. (**b**) Dependence of the obtained fluorescence lifetime on the applied pressure for code D. Integration time range *θ* = 50 ns, around 1000 beads/measurement, photon count numbers approx. between 50 and 160 per bead. An increased pressure, and therefore a higher flow speed and a shorter interaction time, results in slightly longer measured lifetimes. However, the standard deviation of the measured distributions is much larger than the variation due to the flow speed.

Lifetime distributions measured with our LT-FCM setup using neutral density filters of different transmittance in the detection channel are shown in Fig. 6(a), exemplarily for lifetime code B. All other parameters (integration time range, bin width) were kept constant. Increasing signal intensity leads to a shift of the mean measured lifetime to higher values. From a theoretical point of view, the photon count number is not expected to affect the calculated mean lifetime, cf. Fig. 5(a). Thus, according to Fig. 5(b), the differences in filter transmittance seem to vary the *SBR*. However, an increase in filter transmittance should rather improve the *SBR* and thus result in decreasing lifetime values instead of the experimentally observed increase. Therefore, it is more likely that the arriving photon flux exceeds the linear range of the detector and thereby causes the deviation. Considering a comparatively long interaction time of 25 μs results in 125 laser pulses per object. After each pulse there is a time span of 60 ns for photon detection adding up to 7.5 μs of effective photon collection time per event. In turn, assuming 500 photons per event gives one photon every 15 ns on average. The peak intensity at the beginning of the decay may easily be an order of magnitude higher. Despite the low overall number of collected photons, the flux is very high within short time intervals. At such a high flux, the detector is not capable of separately detecting each photon and omits part of the signal. In other words, the detector is operated in the non-linear range. Consequently, the detection efficiency is apparently lower at short delays directly after the pulse and higher at longer delays where the fluorescence intensity is lower. This favours a disproportional accumulation of photons at longer delays. This deformation of the decay curve leads to longer measured lifetimes with increasing signal intensity. In the Supplementary Information we show that the detector is indeed operated in the non-linear range. The effect decreases for lower intensities at the detector and consequently leads to shorter measured lifetimes. This finding clearly demonstrates the challenge of lifetime measurements in flow cytometry with short interaction times. Technical optimization of the LT-FCM setup, such as the use of a more suitable detector, is required to address this issue.

The influence of flow speed, and thus measurement time per bead, is shown in Fig. 6(b). The flow speed was modified by varying the applied pressure. As shown in Fig. 6(b), the measured lifetime increases with increasing pressure. This means, higher flow speed apparently decreases the *SBR* according to Fig. 5(b). The relative increase of the background signal (i.e. reduced *SBR*) is due to the fixed time window used for measurements. For a higher pressure, a bead may already have left the laser spot and the instrument continues measuring background. Therefore, varying flow speed has a potential impact on the lifetime code determination. However, the typical standard deviation in each of these measurements is still three times as large as the obtained variation in measured lifetime values due to pressure variation. Anyway, better stability of lifetime detection could be achieved by dynamic adaption of the measurement time window such that the data acquisition is stopped when the object leaves the laser spot instead of continuously measure for a fixed time window. This would effectively suppress background signal.

### Number of distinguishable codes using the LT-FCM platform

Considering the results from the numerical simulations and instrument characterization, we subsequently modified the measurement parameters to realise optimum discrimination of a set of lifetime-encoded microbeads. The evaluation of the lifetime discrimination capabilities of the LT-FCM was based on the comparison of measured lifetime distributions of samples containing only one type of lifetime-encoded beads with mixtures of these beads.

**Figure 7:**
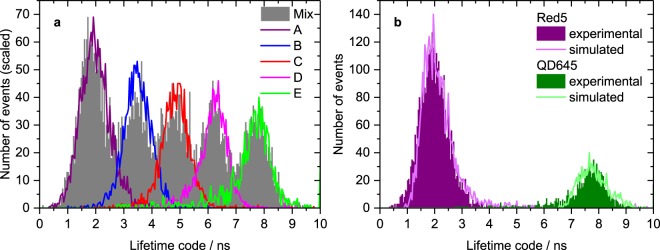
Lifetime distributions from LT-FCM. (**a**) Lifetime distributions of five lifetime codes. The curves with the solid lines were obtained from measurements of pure samples and scaled to the respective peaks in mixture for comparison. Around 1000–4000 beads per code were measured. Photon count numbers range from around 50 to almost 700, integration time range *θ* = 20 ns and an ND filter combination with *T* = 6.3% was used. (**b**) Comparison of experimental and numerical lifetime distributions exemplarily for two codes. Exploiting the lifetimes from ensemble measurements, cf. Table 1, it is possible to numerically generate lifetime distributions that are in good agreement with measured distributions which underlines the applicability of our simulation method.

The resulting distributions for codes A to E are shown in Fig. 7(a). The solid curves represent measurements of single-code samples. The integration time range was *θ* = 20 ns giving the best separation between neighbouring codes according to the results shown in Fig. 3. The peak positions of the mixture and the one-code samples match very well which underlines the good reproducibility.

Even though there is a distinct overlap of the different lifetime distributions which prevents straightforward discrimination, the mean values are still clearly separated. This may not be sufficient for applications which rely on the classification of each single object in a sample such as cell sorting. However, for bead-based assays where only the information whether a certain analyte is contained in a sample or not is needed, the discrimination capabilities might be sufficient. Reducing the experiment to three codes results in completely distinguishable distributions which do not show any significant overlap. The overlap of area-normalized neighbouring distributions then amounts to 2.3% and 7.3%, respectively. Consequently, our LT-FCM setup allows to resolve three codes and we were able to identify up to five lifetime-encoded populations with lifetimes between approximately 2 and 20 ns.

To validate our simulation approach, we took the reference lifetime values from ensemble measurements (cf. Table 1) as notional lifetimes (multi-exponential decays) in simulations and considered the average photon count numbers per bead as well as the standard deviation of the photon count numbers of the LT-FCM measurements. The only free parameter in the simulations was the *SBR*, which was varied to generate simulated distributions mimicking the measured ones. The resulting simulated distributions for the shortest and longest lifetime codes and distributions from LT-FCM shown in Fig. 7(b) match excellently.

## Conclusion

Aiming at the development of a lifetime flow cytometer (LT-FCM) platform consisting of an LT-FCM instrument and encoding luminophores in micrometer-sized beads, we evaluated the impact of measurement parameters and instrument settings (integration time range, data bin width) and measurement conditions (photon count number, signal-to-background ratio) on the detection of luminescence lifetime codes in flow cytometry.

Discrimination of different lifetime codes was assessed by numerical simulations and experimental data from a unique custom-made flow cytometer for time-resolved measurements. Our results reveal the need to carefully optimise measurement parameters. On the one hand, coarse bin width and short integration time ranges, e.g., can lead to systematic deviations of the calculated/measured lifetimes from the reference lifetime values. On the other hand, optimum lifetime discrimination can be realised with a narrow integration time range, as this reduces the otherwise considerable number of background counts that can have a detrimental impact on lifetime measurements with increasing integration time range. Also, higher signal intensities or longer interaction times are advantageous for smaller uncertainties in lifetime code discrimination. The maximum signal intensity, however, is limited by the detector characteristics which restrains the acquirable number of photons to only several hundred events in our setup. Moreover, the ratio of signal and background/noise also influences the mean value of the calculated lifetime. Mean lifetime values measured at high background levels are more erroneous but can give narrower lifetime distributions. Importantly, for LT multiplexing and barcoding only the quality of lifetime discrimination matters and not the measurement uncertainty of lifetime values.

The excellent match between the lifetime distributions calculated by our simulations with measured distributions suggests that this can be exploited for straightforward adjustment of measurement parameters to obtain an improved LT discrimination. The current performance level for lifetime encoding using a single excitation wavelength and a single detection channel with the presented setup is five codes involving organic and inorganic fluorophores. Discrimination capabilities could be further improved by technical modifications, e.g., employing detectors with better dynamic range or flexible adaption of the measurement time window for each object, as well as software upgrades. Presently, on the unique LT-FCM platform described above first assays are being established, using LT-encoded beads and measurements of fluorescence intensities in an additional detection channel for analyte identification and quantification.

## Electronic supplementary material

Supplementary Information

## Data Availability

The datasets generated and analysed during the current study are available from the corresponding author on reasonable request.
