# Supplementary material for: Luminescence lifetime encoding in time-domain flow cytometry
Source: Sci Rep. 2018 Nov 13;8:16715. doi: 10.1038/s41598-018-35137-5 (PMC6233182; doi:10.1038/s41598-018-35137-5)
Supplement: Supplementary file 1 — Supplementary Information [file 41598_2018_35137_MOESM1_ESM.pdf]

Supplementary Information

Luminescence lifetime encoding in time-domain flow cytometry

Daniel Kage[1,3], Katrin Hoffmann[1], Marc Wittkamp[2], Jens Ameskamp[2], Wolfgang Göhde[2], and Ute Resch-Genger[1,*]

[1]BAM Federal Institute for Materials Research and Testing, Biophotonics Division 1.2, Richard-Willstätter-Str. 11, D-12489 Berlin, Germany
[2]Quantum Analysis GmbH, Mendelstraße 17, D-48149 Münster, Germany
[3]Department of Physics, Humboldt-Universität zu Berlin, Newtonstr. 15, D-12489 Berlin, Germany
[*]ute.resch@bam.de

Optical-spectroscopic characterization of lifetime encoding luminophore candidates

Prior to single-particle measurements, we carried out a thorough characterization of the microbeads considered for lifetime encoding. The emission spectra and respective luminescence decay curves of bead ensembles are shown in Fig. S1. As required, all codes can be excited at the same excitation wavelength (488 nm) and detected within the same spectral window, e.g. with a suitable long pass filter, and the decay kinetics differ sufficiently.

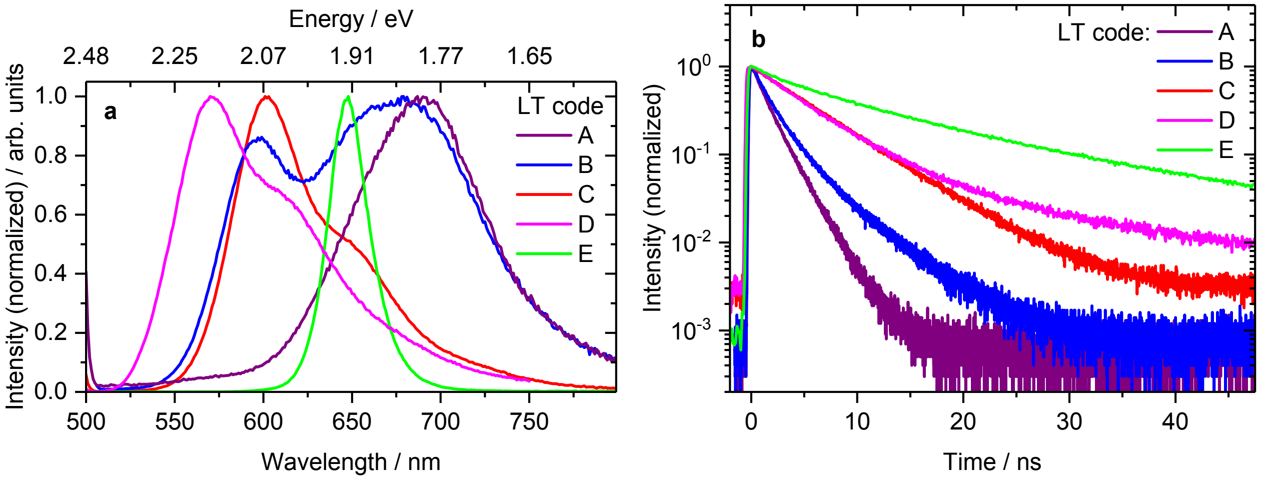

Figure S1. Spectroscopic ensemble measurements. (a) Photoluminescence emission spectra of lifetime codes. λ_{ex} was 488 nm (organic dyes) or 425 nm (quantum dots, for higher signal intensity only). The emission band pass was 4–6 nm. (b) Photoluminescence intensity decay curves of lifetime codes measurements for excitation at 488 nm (16 nm band pass; 5 MHz repetition rate for code E, 10 MHz for all others) and detection with a 645 or 590 nm (sample C, for improved signal intensity only) long pass filter.

Numerical generation of synthetic decay curves

The study of synthetic decay curves is based on pseudo-random numbers following the respective probability distributions. The numerical generation of such distributions will be described in the following based on textbook procedures[1].

Analytical approach for mono-exponential decay

In case of mono-exponential fluorescence decay with lifetime τ, the intensity distribution in time t follows Eq. (S1).

$$I(t) = A\mathrm{e}^{-\frac{t}{\tau}} \tag{S1}$$

Standard pseudo random number generators (PRNG), however, usually provide uniformly distributed random numbers x on the interval $[0,1]$. Thus, we need to map these numbers on a probability distribution that would produce intensity decays according to Eq. (S1). Such a probability distribution is given by Eq. (S2).

$$w(x) = \frac{1}{\tau}\mathrm{e}^{-\frac{t}{\tau}} \qquad \text{fulfilling} \qquad \int_0^\infty \mathrm{d}x\, w(x) = 1 \tag{S2}$$

We now define Eq. (S3),

$$y(x) = \int_0^x \mathrm{d}x'\, w(x'), \tag{S3}$$

which maps $x \in [0,\infty[\to y \in [0,1]$. If Eq. (S3) can be solved for x, we can transform a uniform distribution of values of y to the required distribution of values of x. In case of a mono-exponential decay, cf. Eq. (S1), we have

$$\begin{aligned} y(x) &= \int_0^x \mathrm{d}x \frac{1}{\tau}\mathrm{e}^{-\frac{x'}{\tau}} \\ &= \frac{1}{\tau}(-\tau)\left[\mathrm{e}^{-\frac{x'}{\tau}}\right]_0^x \\ &= 1 - \mathrm{e}^{-\frac{x}{\tau}} \end{aligned}$$

which can be easily inverted to give Eq. (S4).

$$x = -\ln(1-y) \tag{S4}$$

Thus, for mono-exponential decays we can generate uniform distributions of y and transform them to the desired mono-exponential distributions of x, i.e. time t in the physical process.

Von-Neumann rejection for multi-exponential decays

For multiple decay components or non-exponential decays, Eq. (S3) might not be invertible with respect to x. Thus, another solution for the problem must be found. In addition to the now modified $w(x)$ we now need a second distribution $w'(x)$ with $w'(x) > w(x)$ for all x in the required interval. The only requirement is that $w'(x)$ allows for generation of a random number distribution as described above. One then generates pairs of values x_i (distributed according to w') and ξ (distributed uniformly on $[0,1]$). For each pair, the value of x_i is only accepted when

$$\frac{w(x_i)}{w'(x_i)} > \xi \Rightarrow x_i \text{ accepted} \tag{S5}$$

and discarded otherwise. The process is repeated until the desired number of random values has been generated. For multi-exponential decay

$$w(x) = \frac{1}{\sum_j A_j \tau_j} \sum_j A_j \mathrm{e}^{-\frac{x}{\tau_j}} \qquad \text{fulfilling} \qquad \int_0^\infty \mathrm{d}x\, w(x) = 1 \tag{S6}$$

and a suitable choice of w' could be

$$w'(x) = \frac{\sum_j A_j}{\sum_j A_j \tau_j} \mathrm{e}^{-\frac{x}{\tau_\mathrm{m}}} \tag{S7}$$

where $\tau_\mathrm{m} = \max(\tau_j)$. Practically, a loop generates pairs of x_i and ξ and checks the condition of Eq. (S5) each time. The loop terminates when the required number of accepted x_i has been generated.

Numerical simulation of lifetime determination in flow cytometry

Synthetic decay curves generated from pseudo-random numbers as described above can be used as starting point to deduce theoretical dependencies of the measured luminescence lifetime in flow cytometry on various parameters such as integration time range, bin width, signal intensity, or signal-to-background ratio. Therefore, a set of decay curves with certain decay kinetics and given overall number of counts is generated. The data are binned as desired and background counts can be added. Lifetime determination on the simulated data is done by means of

$$\tau_\mathrm{mean} = \frac{\sum_{j=1}^{j_\mathrm{max}} t_j I_j}{\sum_{j=1}^{j_\mathrm{max}} I_j}$$

where t_j is the time value of bin j and I_j is the respective number of counts in that bin. The integration time range is determined by j_max. The number of generated decay curves resembles the number of objects in a flow cytometry experiment and provides statistical relevance of the simulation results. Simulations were performed with custom-made Octave[2] scripts.

Complementary simulation results

In addition to the investigation of the impact of the photon count number on the calculated lifetime for mono-exponential decays without background signal, Fig. S2 shows calculations for bi-exponential decay and background counts. The relative deviation

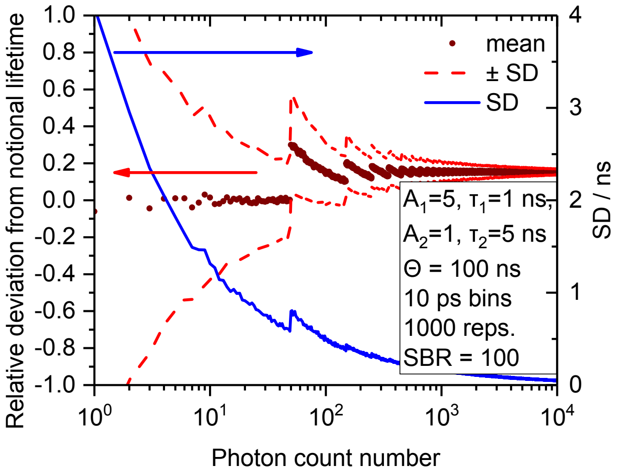

Figure S2. Simulated dependence of the mean lifetime of a bi-exponential decay on the signal-to-background ratio. Integration time range $\theta = 100$ ns, 100 photons, 1000 repetitions (corresponding to 1000 objects in an experiment). The sawtooth-like shape stems from the discrete nature of the background count number. Overall, the mean calculated lifetime is independent of the photon count number.

from the notional lifetime approaches a constant value for higher photon count numbers. The offset from zero deviation is caused by the background counts. The step-like changes in the relative deviation stem from the discrete nature of the counts. Therefore, a background count is, on average, only added when the photon count number exceeds certain values which results in a stepwise change of the simulated *SBR* at small photon count numbers. The discretisation effects vanish for increasing photon count numbers as the background count increase in a more continuous manner. Apart from these artefacts, the relative deviation is independent of the photon count number and therefore also luminophores with bi-exponential (or multi-exponential) decays are expected to be suitable for lifetime encoding.

It was shown in the main manuscript that the *SBR* affects the calculated lifetime. This effect is displayed again for several different notional lifetimes in Fig. S3. Obviously, the relative deviation increases drastically with decreasing notional lifetimes.

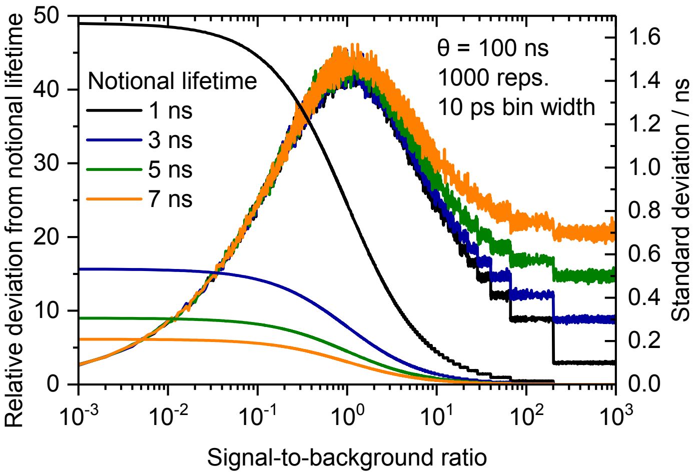

Figure S3. Dependence of deviation of the calculated lifetime from the notional lifetime and the standard deviation on the *SBR* for different values of the notional lifetime. Time range $\theta = 100$ ns, 10 ps bin width, 1000 repetitions (i.e. objects in FCM). As the calculated lifetime approaches $\theta/2$ for very low *SBR* independent of the notional lifetime, the relative deviation is larger for small notional lifetimes lifetimes. The course of the standard deviation is independent of the notional lifetime.

This means, shorter lifetimes undergo a stronger relative distortion due to background counts. Anyway, all measured lifetimes will approach $\theta/2$ with decreasing *SBR*. Consequently, no distinction will be possible beyond a certain background level and lifetime codes may mix upon different *SBR* per code carrier. The standard deviation, however, is basically independent of the notional lifetime for small *SBR* and it is slightly larger for longer notional lifetimes for increasing *SBR*.

As discussed in the main manuscript, the bin width plays an important role for the correct determination of lifetimes. On the contrary, the bin width does not affect the standard deviation, i.e. the precision, of the determined lifetimes as shown in Fig. S4.

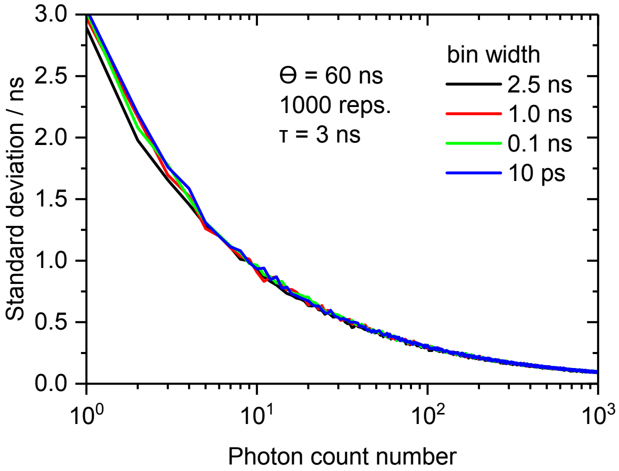

Figure S4. Dependence of the standard deviation of the calculated lifetime on the photon count number for different bin widths. Time range θ was 60 ns, 1000 repetitions were performed (equivalent to 1000 objects in an experiment). Clearly, the bin width does not influence the lifetime distribution standard deviation and is therefore less critical for improved precision.

Analytical expressions for lifetime standard deviation

The analytical expressions for the standard deviation in lifetime estimation procedures used for comparison with our numerical results shall be given here for the sake of completeness. When background is absent, the variance of the lifetime is[3]

$$\mathrm{var}_\tau = \frac{1}{N}\tau^2\frac{k^2}{r^2}\left(1-\mathrm{e}^{-r}\right)\left[\frac{\mathrm{e}^{r/k}\left(1-\mathrm{e}^{-r}\right)}{\left(\mathrm{e}^{r/k}-1\right)^2}-\frac{k^2}{\mathrm{e}^{r}-1}\right],$$

where N is the number of photon counts, τ the actual lifetime, θ the integration time range, k the number of bins, and $r=\theta/\tau$. In case of background signal being present, the expression changes to[3]

$$\mathrm{var}_{\tau,\mathrm{bg}} = -\left[\frac{T^2}{\tau^4}\sum_{i=1}^{k} y_i \frac{\partial^2 \ln y_i}{\partial r^2}\right]^{-1}$$

$$y_i = Np_i = \frac{N}{k}\frac{1}{1-\mathrm{e}^{-r}}\left[b\left(1-\mathrm{e}^{-r}\right)+k(1-b)\left(\mathrm{e}^{\frac{1-i}{k}r}-\mathrm{e}^{-\frac{i}{k}r}\right)\right]$$

$$\frac{\partial^2 \ln y_i}{\partial r^2} = \frac{\mathrm{e}^{r}}{(1-\mathrm{e}^{r})^2}+\frac{-b\mathrm{e}^{-r}+k(1-b)\left[\left(\frac{1-i}{k}\right)^2\mathrm{e}^{\frac{1-i}{k}r}-\left(\frac{i}{k}\right)^2\mathrm{e}^{-\frac{i}{k}r}\right]}{k(1-\mathrm{e}^{-r})p_i}-\left[\frac{b\mathrm{e}^{-r}+k(1-b)\left(\frac{1-i}{k}\mathrm{e}^{\frac{1-i}{k}r}-\mathrm{e}^{-\frac{i}{k}r}\right)}{k(1-\mathrm{e}^{-r})p_i}\right]^2.$$

Technical characteristics of the lifetime flow cytometer

Description of optical components

A detailed list of the optical filters and mirrors of the lifetime flow cytometer (LT-FCM) is given in Tab. S1.

Element	Optical characteristics
M	Mirror
DM1	Dichroic mirror (620 nm)
ND	Neutral density filter
LP1	Long pass (630 nm)
DM2	Dichroic mirror (500 nm)
LP2	Long pass (435 nm)
DM3	Dichroic mirror (538 nm)
BP	Band pass ((520 ± 14) nm)
BS	Beam splitter (90:10)
LP3	Long pass (530 nm)

Table S1. List of optical elements in the LT-FCM.

Linearity of the detection system

Operating a detection system outside its linear range leads to signal distortion. Thus, we assessed the response of our detection system in terms of photon counts as a function of the transmittance of the used neutral density filter transmission, see Fig. S5. Even though the number of acquired photons is low on the scale of typical time-resolved measurements, the detector is already

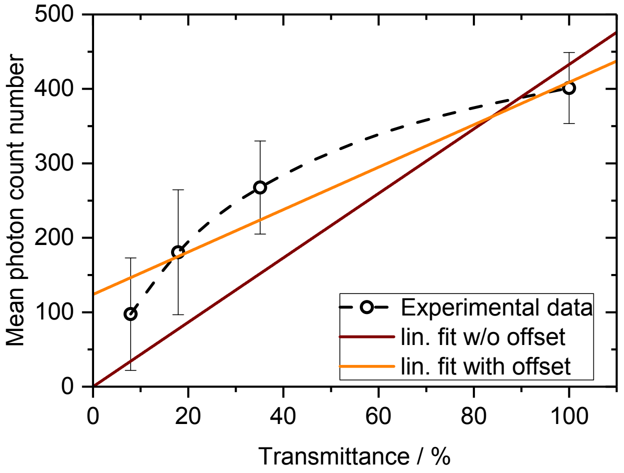

Figure S5. Photon count number within the time region of decay after the laser pulse as a function of the filter transmittance. A linear dependence of the photon count number on the filter transmittance was expected. Linear fits provide visualization of the deviation from linearity. Consequently, the detector is operated beyond its linear range due to too high signal intensity.

operated beyond the linear range. This is due to the very short interaction time in flow cytometry measurements and even for the lowest transmittance tested here the signal intensity is still critical. The two linear fits to the data points illustrate the deviation from linearity. The fit including an offset parameter represents the assumption of a background signal independent of the filter transmittance, i.e., electronic noise. The fit without such an offset neglects a background signal. As the measurement time per object in flow cytometry has to be short by concept of the technique, it is unavoidable to find a trade-off between the number of accumulated photons and the detector linearity.

References

1. Koonin, S. E. *Computational Physics* (The Benjamin/Cummings Publishing Company, Inc., 1985).

2. Eaton, J. W., Bateman, D., Hauberg, S. & Wehbring, R. *GNU Octave version 4.2.2 manual: a high-level interactive language for numerical computations* (2018).

3. Köllner, M. & Wolfrum, J. How many photons are necessary for fluorescence-lifetime measurements? *Chem. Phys. Lett.* **200**, 199–204, DOI: 10.1016/0009-2614(92)87068-z (1992).
